# 

**Published:** 1892-04

**Authors:** 


					﻿The American Obstetrical Society will hold its next
regular meeting in the Hahnemann Medical College building,
Fifteenth Street, above Race, Philadelphia, on April 20th, at
eight o’clock p. m. Papers are expected from Drs. Geo. B.
Peck, Providence, R. I.; W. C. Dake, Nashville, Tenn.;
Charles B. Gilbert, Washington, D. C.; Loomis L. Danforth,
New York; A. R. Thomas, and J. Nicholas Mitchell, Phila-
delphia. A cordial invitation is extended to all members of the
profession interested in this important specialty to attend with-
out further notice. Dr. Thomas Franklin Smith, 264 Lenox
Avenue, New York, is chairman of the Board of Censors. Ap-
plications for membership may be sent to him. Any regular
graduate in medicine in good standing, a practitioner of Homoe-
opathy, is eligible for membership. The annual dues are one
dollar. There is no initiation-fee. Further information in re-
gard to the society may be obtained if desired by addressing
the President, Dr. George William Winterburn, No. 328 West
Twenty-first Street, New York.
NOTICE.
All articles for publication, books for review, business communications, etc.,
must be sent to Editor, 1125 Spruce Street, Philadelphia.
Subscribers will please notice that this Journal will be sent until arrears
are paid and it is ordered discontinued.
				

